# Failure of the dog culling strategy in controlling human visceral leishmaniasis in Brazil: A screening coverage issue?

**DOI:** 10.1371/journal.pntd.0007553

**Published:** 2019-06-26

**Authors:** Lucas Christian de Sousa-Paula, Lidiane Gomes da Silva, Kamila Gaudêncio da Silva Sales, Filipe Dantas-Torres

**Affiliations:** 1 Department of Immunology, Aggeu Magalhães Institute, Oswaldo Cruz Foundation, Recife, Pernambuco, Brazil; 2 Centro Universitário do Vale do Ipojuca (UNIFAVIP/Wyden), Caruaru, Pernambuco, Brazil; Vienna, AUSTRIA

## Abstract

In the present study, we assessed the annual screening coverage (i.e., the percentage of dogs that are screened for anti-*Leishmania* antibodies annually) in the municipality of Sobral, Ceará state, Brazil. Data on the number of dogs screened during 2008−2017 (except 2010) were obtained from the Centre for Zoonoses Control of Sobral. The annual screening coverage during 2012−2017 was calculated. Data on human visceral leishmaniasis (VL) cases during 2008−2017 were compiled from the National Disease Notification System. Correlation analyses were performed to assess the correlation between canine and human data. During 2008−2017, 73,964 dogs (range, 0 to 13,980 dogs/year) were serologically screened and 2,833 (3.8%) were positive. The annual screening coverage during 2012−2017 ranged from 11.1% to 45.7%. There were no significant correlations between the number of dogs culled and the number of human VL cases, canine positivity and human VL incidence, number of dogs culled and human VL incidence, or between canine positivity and number of human VL cases. An inconsistent and relatively low annual screening coverage was found in the study area, with no dog being screened in 2010 due to the lack of serological tests. Our results highlight that many dogs potentially infected with *Leishmania infantum* have been virtually overlooked by public health workers in the study area, perhaps with a negative, yet underestimated, impact on the control of canine and human VL. Hence, the failure of the dog culling strategy in controlling human VL in Brazil may be due to the low screening coverage and low percentage of culled dogs, rather than the absence of associations between canine and human infections.

## Author summary

The euthanasia of *Leishmania*-seropositive dogs has been recommended for controlling human visceral leishmaniasis (VL) in some countries where this zoonosis is endemic. We assessed the annual screening coverage (i.e., the percentage of dogs living in a given area that are screened for anti-*Leishmania* antibodies annually) in the municipality of Sobral, Ceará state, one of the main foci of human VL in Brazil. From 2008 to 2017, nearly 74,000 dogs were screened and 3.8% of them were positive for anti-*Leishmania* antibodies. No statistically significant correlation was found between the number of dogs culled annually and the incidence of human VL. The annual screening coverage ranged from 11.1% to 45.7%. Our results highlight an inconsistent and relatively low annual screening coverage, indicating that dogs potentially infected with *L*. *infantum* have been virtually overlooked by public health workers in the study area.

## Introduction

Human visceral leishmaniasis (VL) is a neglected vector-borne disease of great public health significance. The disease is endemic in more than 60 countries, with estimated 200,000 to 400,000 human cases and 20,000 to 40,000 deaths occurring annually worldwide [1]. In the Americas, VL is a zoonosis caused by *Leishmania infantum* and Brazil concentrates most of the notified cases, with estimated 4,200 to 6,300 new cases per year [1].

*Leishmania infantum* is transmitted to susceptible hosts, including humans, through the bite of infected female phlebotomine sand flies [2]. While several animals can serve as a source of infection to phlebotomine sand flies, dogs are the most important reservoirs in domestic settings [3]. As such, the presence of infected dogs is reputed to be a risk for *L*. *infantum* infection in humans [4].

In this perspective, the culling of *Leishmania*-seropositive dogs has been recommended as a control measure in many countries where human VL is endemic, including in Brazil [5]. Currently, this measure is one of three main strategies of the VL surveillance and control program of the Ministry of Health of Brazil, which also includes early diagnosis and treatment of human cases, as well as vector control [6]. Nonetheless, the dog culling strategy has long been an issue of debate, as there is no convincing scientific evidence supporting its effectiveness [5,7,8].

Reasons for the failure of the dog culling strategy in controlling human VL in Brazil may include limited sensitivity of serological tests, long delay between diagnosis and the removal of infected dogs, and rapid replacement of culled dogs by new susceptible ones [5,9–13]. Another important factor that may negatively influence the effectiveness of the dog culling strategy is the annual screening coverage, i.e., the percentage of dogs living in a given area that are screened for anti-*Leishmania* antibodies annually. In a recent study conducted in the city of Araçatuba, south-eastern Brazil, the authors reported an annual screening coverage ranging from 1.0% to 10.0% [14]. This study highlighted that the effectiveness of the dog culling strategy is likely compromised by the low annual screening coverage.

In the present study, we assessed the annual screening coverage in the municipality of Sobral, a historical focus of human VL in north-eastern Brazil, where the first outbreak of the disease was detected in this country and where the dog culling strategy was firstly implemented [15]. In particular, our hypothesis was that the inconsistent annual screening coverage may be one of the reasons for the failure of the dog culling strategy in controlling human VL in an important urban focus of this disease in Brazil.

## Methods

### Study area

**Fig 1:**
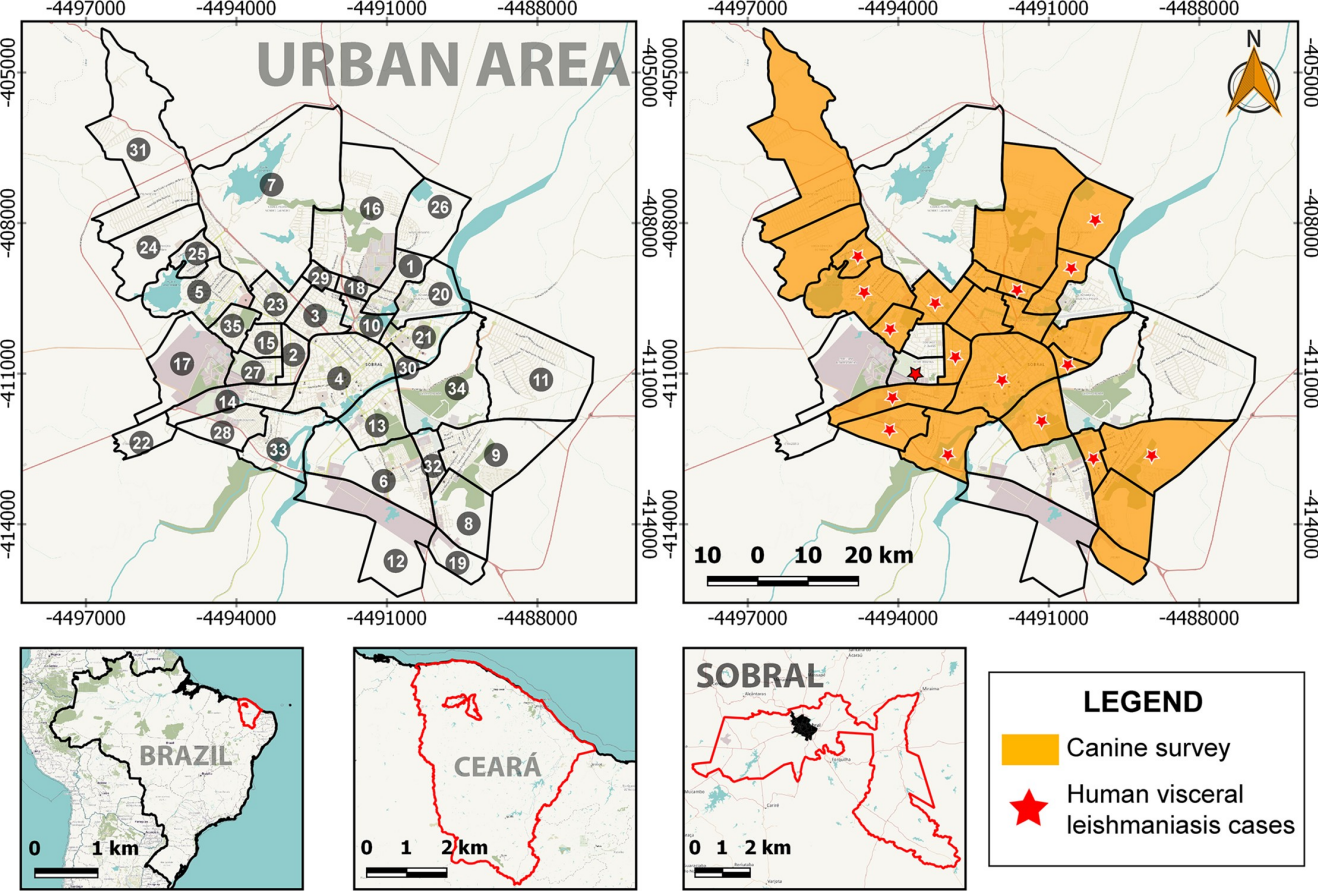
Districts where dog were screened and where human visceral leishmaniasis were notified in the urban area of Sobral, Ceará state, Brazil, 2008–2017. Sobral urban area divided into 35 districts. (1) Alto da Brasília, (2) Alto do Cristo, (3) Campo dos Velhos, (4) Centro, (5) Cidade Dr. José Euclides Ferreira Gomes, (6) Cidade Gerardo Cristino de Menezes, (7) Cidade Pedro Mendes Carneiro, (8) Cohab I, (9) Cohab II, (10) Coração de Jesus, (11) Distrito Industrial, (12) Dom Expedito, (13) Dom José, (14) Domingos Olímpio, (15) Edmundo Monte Coelho, (16) Expectativa, (17) Jatobá, (18) Jerônimo de Medeiros Prado, (19) Jocely Dantas de Andrade Torres, (20) Juazeiro, (21) Junco, (22) Juvêncio de Andrade, (23) Mucambinho, (24) Nossa Senhora de Fátima, (25) Nova Caiçara, (26) Novo Recanto, (27) Padre Ibiapina, (28) Padre Palhano, (29) Parque Silvana, (30) Pedrinhas, (31) Renato Parente, (32) Sinhá Sabóia, (33) Sumaré, (34) Várzea Grande, (35) Vila União. Maps were produced using QGIS based on public geographic data from OpenStreetMap.

The municipality of Sobral (03°40'26"S, 40°14'20"W, altitude: 70 m above the sea level) is located in the north-western region of Ceará state, 240 km away from Fortaleza (the capital city). Sobral is home to 205,529 residents spread over an area of 2,122.989 km^2^ (including both rural and urban areas). Its urban area is divided into 35 districts (Fig 1). Sobral has the second best human development index (HDI = 0.714) of Ceará and 75.6% of its territory has adequate sanitary sewers [16]. The climate is tropical semi-arid (steppe climate), characterized by rainy and dry periods, with rains concentrated from January to May, monthly relative humidity ranging from 56.2% to 85.9% and monthly temperature ranging from 21°C to 39°C [17].

### Data sources

Data regarding the number of dogs serologically screened and the number of seropositive ones were obtained from the Centre for Zoonoses Control (CZC) of Sobral. Data from 2008 to 2017 (except 2010, when no screening was conducted due the lack of serological tests) and from 25 out of 35 districts were obtained, representing 71.4% of urban area of Sobral (there was no screening in 10 districts due to the CZC’s logistical reasons). At the CZC, dogs are serologically screened when the owners spontaneously bring their dogs to for testing or when public health agents visit each district to sample and screen both resident and stray dogs. Until 2009, all dogs were screened using an indirect fluorescent antibody test (IFAT) (IFI—Leishmaniose Visceral Canina, Bio-Manguinhos, Fiocruz, Rio de Janeiro, Brazil). Since 2011, all dogs started to be screened using a rapid immunochromatographic test (ICT) (TR DPP Leishmaniose Visceral Canina, Bio-Manguinhos, Fiocruz, Rio de Janeiro, Brazil) and, if positive, retested using an enzyme-linked immunosorbent assay (ELISA) (EIE—Leishmaniose Visceral Canina, Bio-Manguinhos, Fiocruz, Rio de Janeiro, Brazil). The CZC informed that all dogs positive by IFAT (until 2009) and by both ICT and ELISA (from 2011 onwards) were humanely culled and then the carcasses were incinerated, as recommended by the Ministry of Health of Brazil [6].

Secondary data regarding human VL cases notified during 2008−2017 were obtained from Health Surveillance Secretariat of Sobral. Data were compiled from the National Disease Notification System (SINAN) database and processed anonymously.

Data regarding the human population size during 2008–2017 were obtained from Brazilian Institute of Geography and Statistics (IBGE) [16]. The canine population size in the last six years (2012–2017) were obtained from CZC, which conducts annual censuses in Sobral.

The maps were produced using QGIS software version 2.18.28 [18] and based on public geographical data obtained from OpenStreetMap [19].

### Statistical analyses

The annual screening coverage was calculated by dividing the number of dogs serologically screened in a given year by the canine population size in the same year and then multiplied by 100. Positivity was calculated by dividing the number of seropositive dogs by the number of dogs serologically screened and then multiplied by 100. Results were expressed as percentages. Incidence of human VL was calculated by dividing the number of new cases in a given year by the human population size in the same year and multiplied by 100,000. Results were expressed as the number of cases per 100,000 population.

Prior to statistical analyses, normality of data was checked using Lilliefors. As data presented a non-normal distribution, the correlation between dog culling and human VL incidence was investigated using Spearman’s coefficient (*rs*). The trend in the human VL incidence in Sobral over the years was assessed using Mann-Kendall trend test. The differences were considered statistically significant when *P* ≤ 0.05. Statistical analyses were performed using BioEstat v. 5.3 (Instituto Mamirauá, Tefé, Amazonas, Brazil) and PAST 3.23 [20].

### Ethics statement

The Health Secretary of Sobral (0184/2018) and Research Ethics Committee (97934718.4.0000.5190) of the Aggeu Magalhães Institute (Fiocruz) approved the access and using of secondary data (number of dogs serologically screened and human VL cases) analysed in this research.

## Results

### Dog culling

From 2008 to 2017, 73,964 dogs were serologically screened for anti-*Leishmania* antibodies, with an average of 8,218 dogs sampled per year (range, 0–13,980). The annual screening coverage from 2012 to 2017 ranged from 11.1% to 45.7% (Table 1). In total, 2,833 out of 73,964 dogs serologically screened resulted positive, representing an overall positivity of 3.8%. The annual positivity ranged from 0.5% to 8.1% (Table 1). There was no correlation between the annual screening coverage and the number of seropositive dogs detected each year (*rs*_(4)_ = 0.486; *p* = 0.3287).

**Table 1:** Overview of human visceral leishmaniasis and dogs screened for *Leishmania*-antibodies in Sobral, Ceará state, Brazil, 2008–2017.

Year	Canine population ^a^	Dogs screened	Screening coverage	Positive dogs (%)	Human population	Human cases	Incidence (per 100,000 population)
2008	-	10,237	-	187 (1.8%)	180,046	35	19.4
2009	-	3,239	-	15 (0.5%)	188,271	35	18.6
2010	-	0	-	-	188,233	38	20.2
2011	-	13,980	-	309 (2.2%)	190,724	49	25.7
2012	25,037	4,518	18.0%	189 (4.2%)	193,134	29	15.0
2013	25,882	11,822	45.7%	957 (8.1%)	197,663	27	13.7
2014	27,395	3,032	11.1%	229 (7.5%)	199,750	11	5.5
2015	29,017	9,186	31.7%	571 (6.2%)	201,756	13	6.4
2016	31,937	8,283	25.9%	159 (1.9%)	203,682	9	4.4
2017	31,937	9,667	30.3%	217 (2.2%)	205,529	1	0.5
Total	-	73,964	-	2,833 (3.8%)	-	247	-

^a^ Canine population size in the last six years (2012–2017) obtained from annual censuses in Sobral conduct by the Centre for Zoonoses Control.

**Fig 2:**
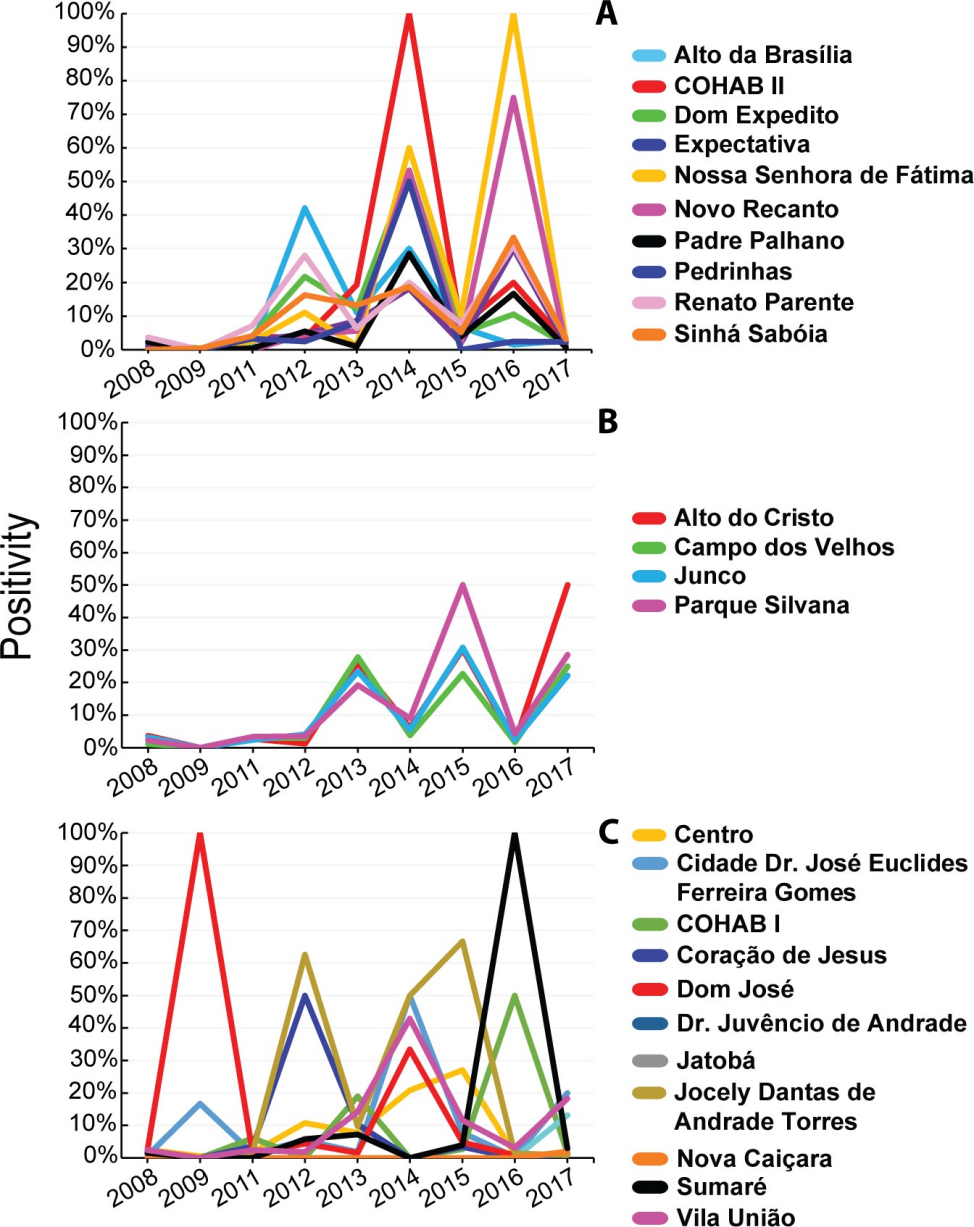
Positivity for anti-*Leishmania* antibodies in dogs from districts of Sobral (Ceará state, Brazil), from 2008 to 2017. (A) Districts displaying bimodal peaks in even-years. (B) Districts displaying bimodal peaks in odd-years. (C) Districts displaying no defined pattern.

Over the study period, the decrease in the canine positivity in a given year was usually preceded by a higher positivity in the previous year, resulting in a bimodal pattern, with peaks of positivity every two years in some districts (Fig 2). This bimodal pattern was observed in most districts (14/25) (Fig 2A and 2B), with the remaining districts displaying no defined pattern (Fig 2C).

The positivity in each district ranged from 1.6% to 13.1%. In average, 113 dogs were culled per district (range, 1–303 dogs/district) during the study period. In addition, nine out of 25 districts showed positivity above average (Table 2).

**Table 2:** Human visceral leishmaniasis cases and dogs screened for anti-*Leishmania* antibodies in the districts of Sobral, Ceará state, Brazil, 2008–2017.

Districts	Human cases	Dogs screened	Positive dogs	Positivity
Alto da Brasília	10	4,405	165	3.7%
Alto do Cristo	4	5,850	207	3.5%
Campo dos Velhos	0	3,122	96	3.1%
Centro	29	8,827	274	3.1%
Cidade Dr. José Euclides Ferreira Gomes	52	9,032	255	2.8%
COHAB I	0	794	43	5.4% ^a^
COHAB II	3	3,557	232	6.5% ^a^
Coração de Jesus	0	467	18	3.9%
Dom Expedito	11	5,790	303	5.2% ^a^
Dom José	8	5,093	122	2.4%
Dr. Juvêncio de Andrade	0	99	13	13.1% ^a^
Expectativa	14	3,045	104	3.4%
Jatobá	0	62	1	1.6%
Jocely Dantas de Andrade Torres	0	632	27	4.3%
Junco	19	3,974	160	4.0%
Nossa Senhora de Fátima	0	389	24	6.2% ^a^
Nova Caiçara	1	209	4	1.9%
Novo Recanto	1	1,742	52	3.0%
Padre Ibiapina	14	-	-	-
Padre Palhano	19	4,311	76	1.8%
Parque Silvana	0	1,707	94	5.5% ^a^
Pedrinhas	2	988	30	3.0%
Renato Parente	0	844	62	7.3% ^a^
Sinhá Sabóia	26	2,716	154	5.7% ^a^
Sumaré	16	3,120	104	3.3%
Vila União	18	3,189	213	6.7% ^a^
Total	247	73,964	2,833	3.8%

^a^ Districts that had displayed above average positivity.

### Human VL

**Fig 3:**
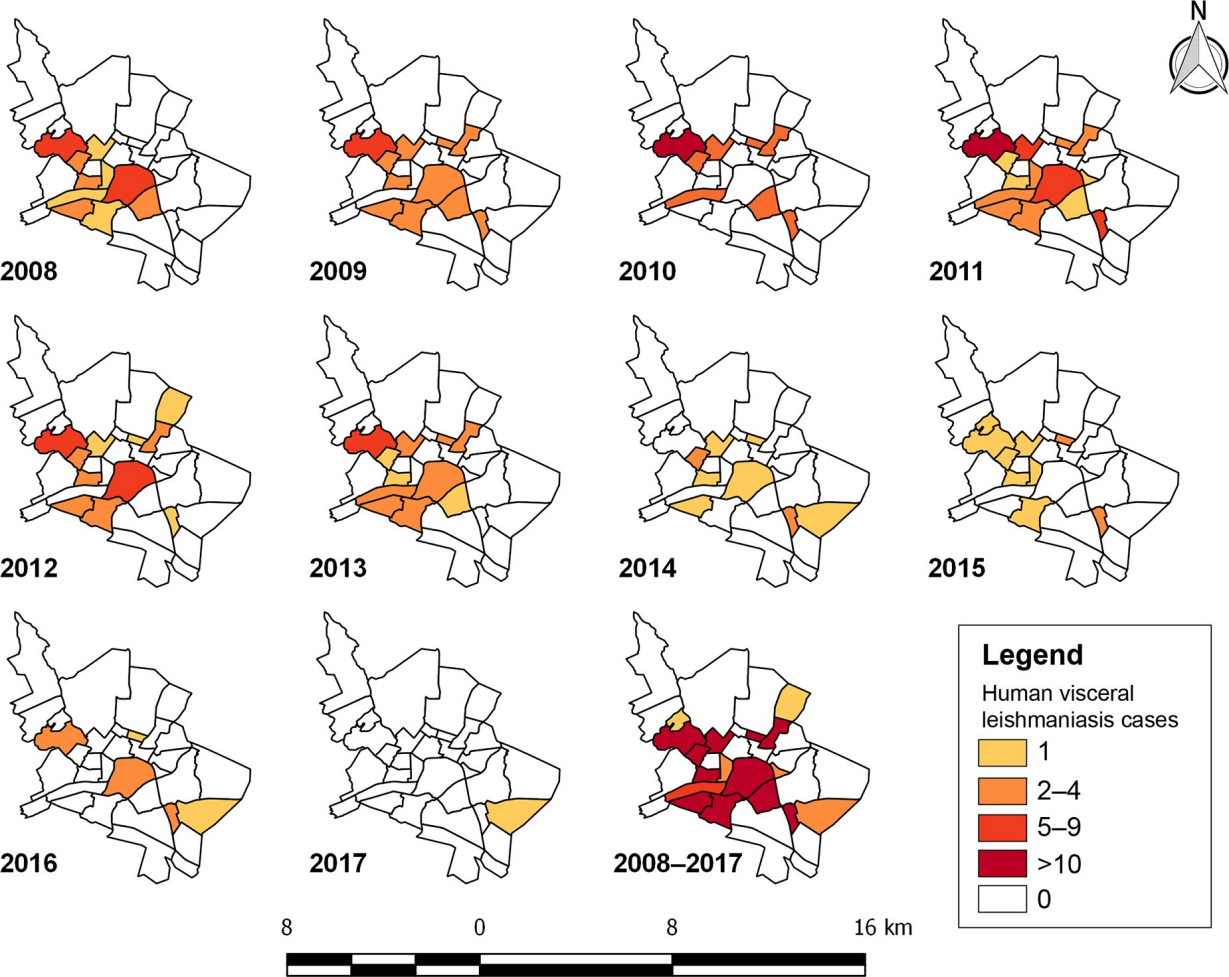
Human visceral leishmaniasis cases notified in districts of Sobral, Ceará state, Brazil, 2008–2017. Maps were produced using QGIS.

From 2008 to 2017, 247 human cases of VL were notified in 17 districts of Sobral. Although the Padre Ibiapina district reported 14 human VL cases, no single dog was serologically screened in this district during the same period (Fig 1 and Table 2). The total number of cases per district ranged from one to 52. In addition, 11 districts reported at least 10 cases from 2008 to 2017 (Table 2 and Fig 3).

The incidence of human VL in Sobral ranged from 0.5 to 25.7 cases per 100,000 population (Table 1) during the study period and no fatal cases were recorded. There was a significant decreasing trend in the human VL incidence in Sobral from 2008 to 2017 (Mann-Kendall trend test, *p*  =  0.001).

### Correlation between dog culling and human VL

There were no statistically significant correlations between the number of dogs culled and the number of human VL cases (*rs*_(7)_ = –0.367; *p* = 0.332), canine seropositivity and human VL incidence (*rs*_(7)_ = –0.067; *p* = 0.864), number of dogs culled and human VL incidence (*rs*_(7)_ = –0.050; *p* = 0.898), or between canine seropositivity and number of human VL cases (*rs*_(7)_ = –0.377; *p* = 0.318).

## Discussion

An overall positivity of 3.8% was calculated in the present study, considering data obtained from nearly 74,000 dogs, which were serologically screened from 2008 to 2017 in Sobral. This value is similar to that reported in the same municipality in the 1950s when skin and liver biopsies were analysed [21]. On the other hand, our results are lower than the average seropositivity (i.e., 6.7%) reported for Ceará state [22]. Indeed, VL has focal distribution pattern and combining data from different districts or municipalities may cause a dilution effect, potentially masking the local reality. For instance, a study conducted in Fortaleza revealed that 21.4% (135/631) of the stray dogs and 26.2% (197/750) of the domestic dogs were seropositive [23]. A recent study using real-time PCR for detecting *Leishmania* DNA in dogs from Sobral reported a positivity of 36.8% [24]. Altogether, these results may suggest that the actual infection rate in dogs in Sobral may be underestimated when only serological tests are used, in a similar fashion to what has been observed in other endemic areas [25]. The fact that control measures eliminate only seropositive dogs (thus not all infected dogs) has been suggested as one of the reasons for the failure of the dog culling strategy in controlling *L*. *infantum* infection in dogs and, therefore, in humans [26].

The annual positivity in dogs found herein showed a bimodal pattern in most of the districts (14/25) over the study period. In other words, there was a fluctuation in the positivity, alternating between one year of high and another of relatively low positivity. This finding may raise interesting questions concerning the possible determinant factors for this temporal pattern. Considering that phlebotomine sand flies are present during the whole year in Sobral [27,28], this temporal pattern is probably not related to a possible vector-related transmission pattern. The absence of a well-defined fluctuation pattern in human VL cases also reinforces this hypothesis.

Another important question is why the positivity did not present a decreasing trend over the years, considering that every year thousands of dogs were serologically screened and positive ones culled. This may be related to many factors, but may be partly explained by the inconsistent and relatively low annual screening coverage observed throughout the study period, always below 50%. These data show that thousands of dogs were virtually overlooked by public health authorities, with a potential negative impact on control measures against human VL in the study area. Nonetheless, the actual impact of the low annual screening coverage in the control of human VL is likely to be currently underestimated. A mathematical model suggested that the dog culling could be effective in reducing the proportion of infected dogs and humans if 70–90% of the seropositive dogs are culled [29]. In turn, the percentage of dogs that are *de facto* serologically screened annually reported in different studies hardly ever surpass 50% [14,30, present study]. Furthermore, our data indicated no correlation between screening coverage and number of seropositive dogs detected, thus culled. This suggests that a higher annual screening coverage does not necessarily mean that more infected dogs will be detected and culled. This lack of correlation may also be linked to inherent limitations of serological tests currently used in Brazil [31].

The high cost of the human VL control program leads to the discontinuity of the activities (e.g., serological screening), especially during dengue epidemics [14]. Indeed, the human VL control program generally uses the same human and financial resources that are administered for the control of other diseases in the municipality [14]. As a consequence, even if theoretically the dog culling strategy could be successful in a perfect scenario (i.e., all presently and newly infected dogs are eliminated on a systematic manner), in practice, the VL control program will rarely manage to achieve the requirements for a sustainable successful outcome.

In addition to the low annual screening coverage, another important factor to be considered is the time frame between exposure to infected phlebotomine sand flies and the production of anti-*Leishmania* antibodies in dogs. Indeed, it may take up to two years after infection for a dog to produce detectable antibodies [26,32]. Thus, in practice, many seronegative dogs may actually be already infected; they just have not seroconverted yet. And, if these dogs are not detected in a given annual screening, they may also remain for months or years serving as a source of parasites to the vectors.

Cases of human VL have been reported annually in Sobral [33,34], confirming that this municipality remains as an important focus of human VL in Brazil. In the current study, there was a statistically significant decreasing trend in the incidence of human VL in Sobral from 2008 to 2017. Interestingly, this decreasing trend was observed in five out of six municipalities of Ceará that reported over 100 human VL cases from 2008 to 2017, namely Fortaleza, Caucaia, Sobral, Maracanaú, and Juazeiro do Norte [34]. It remains, however, unclear whether this decreasing trend in human VL incidence in some municipalities of Ceará is a result of control strategies (i.e., dog culling, vector control and/or early treatment of human cases) or a natural temporal pattern of the disease in the area, which may be governed by yet unknown factors.

Although some Brazilian studies suggested that dog culling associated with other strategies (e.g., residual spraying and treatment of human cases) could help controlling human VL (e.g., [35,36]), a systematic review, which was based on an evidence report that was requested by the Pan American Health Organization, concluded that well-designed intervention studies are scarce and that routine control strategies against the canine reservoirs are based on weak and conflicting evidence [7]. Indeed, there has been much debate about the ethical aspects and the effectiveness of the dog culling strategy and the need for alternative strategies has been widely discussed [5,8,37–39]. For instance, the effectiveness of the community-wide application of collars impregnated with pyrethroids on dogs in reducing the risk of infection in these animals has been demonstrated in several laboratory and field studies [40–42]. In fact, even if there may be obvious operational difficulties in the relation to the large-scale use of dog collars (e.g., collar losses), this strategy is acknowledged to be most effective in reducing the infection risk in dogs and may eventually reduce the risk of infection in humans and human VL incidence [43,44]. Other strategies such as vaccination, preventive use of immune modulator drugs (e.g., domperidone), and treatment of sick dogs with leishmanicidal and leishmaniostatic drugs are nowadays available, but should be recommended by veterinarians on a case-by-case basis [45].

### Conclusion

In conclusion, we found an inconsistent and relatively low annual screening coverage in the study area, with no dog being screened in 2010 due to the lack of serological tests. Our results highlight that many dogs potentially infected with *L*. *infantum* have been virtually overlooked by public health workers in the study area, perhaps with a negative, yet underestimated, impact on the control of canine and human VL. Hence, the failure of the dog culling strategy in controlling human VL in Brazil could also be, in some instances, a result of the low screening coverage and low of percentage of culled dogs, rather than the absence of an association between canine and human infections.
